# Editorial: Multifaceted Interactions Between Immunity and the Diseased Brain

**DOI:** 10.3389/fncel.2022.941590

**Published:** 2022-06-08

**Authors:** Kristen E. Funk, Axel Montagne, Ana M. Falcao, Sandro Da Mesquita

**Affiliations:** ^1^Department of Biological Sciences, University of North Carolina at Charlotte, Charlotte, NC, United States; ^2^UK Dementia Research Institute, Edinburgh Medical School, University of Edinburgh, Edinburgh, United Kingdom; ^3^Centre for Clinical Brain Sciences, University of Edinburgh, Edinburgh, United Kingdom; ^4^Laboratory of Molecular Neurobiology, Department Medical Biochemistry and Biophysics, Biomedicum, Karolinska Institutet, Stockholm, Sweden; ^5^Life and Health Sciences Research Institute (ICVS), School of Medicine, University of Minho, Braga, Portugal; ^6^Department of Neuroscience, Mayo Clinic, Jacksonville, FL, United States

**Keywords:** immune response, glial cells, brain borders, neuroinflammation, neurodegeneration

Neuroimmune interactions are closely entangled with different aspects of tissue physiology and dysfunction. This Research Topic is a compilation of five review and three original articles that mainly focus on different neuroimmune cellular and molecular players and their roles in central nervous system (CNS) health and disease.

Immune cells circulating in the blood, residing in the meninges and choroid plexus, as well as those inhabiting the CNS actively participate in tissue surveillance and regulate neural function and behavior (Derecki et al., 2010; Filiano et al., 2016; Alves de Lima et al., 2020a; Croese et al., 2021; Da Mesquita et al., 2021a; das Neves et al., 2021). Lutshumba et al. are contributing with a manuscript entitled “*Dysregulation of systemic immunity in aging and dementia*,” which broadly summarizes the age-related changes in peripheral immune function, focusing on adaptive immune cells as a source of inflammation, and discusses how untamed lymphocyte activation may exacerbate both systemic and CNS inflammation, ultimately contributing to the development of dementia. Still centering on peripheral immune responses in neurodegeneration, the manuscript “*WHOPPA enables parallel assessment of LRRK2 and GCase enzymatic activity in Parkinson's disease monocytes*,” by Wallings et al. proposes an optimized protocol for the collection, processing, and analysis (by flow cytometry) of peripheral blood mononuclear cells from idiopathic Parkinson's disease patients, and healthy controls, coined “*WHOPPA*.” In this study, the authors suggest that this method can be used, amongst other things, for the standardized assessment of the levels of leucine-rich repeat kinase 2 (LRRK2) and glucocerebrosidase (GCase) in blood monocytes of healthy or idiopathic Parkinson's disease patients, which might be promising and reliable biomarkers.

Many interactions between the CNS and immune cells take place in the vicinity of the blood-brain barrier (BBB) and are modulated by the cellular components of the neurovascular unit, such as the tightly bound blood endothelial cells, perivascular macrophages, pericytes, and the ensheathing astrocytic endfeets (Sweeney et al., 2019; Alves de Lima et al., 2020b; Croese et al., 2021; Procter et al., 2021). The BBB, formed by the blood endothelial cells, is at the center of the study by Dayton et al. “*Expression of IL-20 receptor subunit beta is linked to EAE neuropathology and CNS neuroinflammation*.” Overall, the data in this manuscript indicate that an upregulation of interleukin 20 (IL-20) receptor subunit beta in the neurovasculature of mice with experimental autoimmune encephalomyelitis might be upstream of C-X-C motif chemokine ligand 12 mediated BBB disruption and contribute to immune cell extravasation into the CNS.

Under healthy conditions, brain border tissues like the meninges and the choroid plexus stroma harbor a variety of immune cells that are physically separated from the brain parenchyma. In certain diseases, however, these neuroimmune interfaces may serve as gateways for peripheral immune cell entry into the CNS (Alves de Lima et al., 2020b; Croese et al., 2021; Cui et al., 2021; Da Mesquita et al., 2021a; Rustenhoven et al., 2021). In fact, a study focusing on the choroid plexus by Van Hoecke et al. shows that mice deficient in the Niemann-Pick disease type C intracellular cholesterol transporter 1 (*Npc1*) gene show an exacerbated inflammatory response at this barrier tissue that is accompanied by autophagosome formation in choroid plexus epithelial cells and presence of enlarged extracellular vesicles in the cerebrospinal fluid (CSF). In this study entitled “*Involvement of the choroid plexus in the pathogenesis of Niemann-Pick disease type C*,” the authors also show that the proinflammatory extracellular vesicles isolated from the CSF of *Npc1*-deficient mice can *per se* recapitulate the typical gliosis observed in the brains of the Niemann-Pick disease type C mouse model.

Regardless of their often-remote anatomical localization, border-associated immune cells actively secrete cytokines and other soluble factors that support brain physiology by modulating the function of parenchymal neurons and glia. However, when unbalanced or unchecked, the crosstalk between immune and neuronal cells might become detrimental to brain function (Alves de Lima et al., 2020b; Croese et al., 2021). This type of deleterious neuroimmune interactions is thought to be triggered by certain microbial infections (Funk and Klein, 2019; Garber et al., 2019; Funk et al., 2021) and may underly the appearance of neurological disorders, a topic that is thoroughly discussed by Lotz et al. in “*Microbial infections are a risk factor for neurodegenerative diseases*.”

The (re)discovery of a genuine and functional meningeal lymphatic vascular system that constantly drains the brain and spinal cord has also challenged some pre-established concepts of CNS immune privilege and led to new hypotheses regarding the role of lymphatic drainage in brain physiology and disease (Louveau et al., 2015; Da Mesquita et al., 2018, 2021b; das Neves et al., 2021). This Research Topic includes a review manuscript, “*Neuroinflammation-driven lymphangiogenesis in CNS diseases*,” by Hsu et al. where authors highlight the phenomenon of lymphangiogenesis by a subset of meningeal lymphatics near the cribriform plate and closely examine the current knowledge about the roles of these particular meningeal lymphatic vessels in models of neuroinflammatory conditions.

Microglia, the brain-resident innate immune cells, as well as other glial cell populations like astrocytes and oligodendrocyte lineage cells, play a central role not only in supporting neuronal function, but also in the neuroimmune response to pathogenic insults, including trauma, infection, inflammation, accumulation of misfolded proteins and neurodegeneration (Keren-Shaul et al., 2017; Falcao et al., 2018; Wendeln et al., 2018; Castellani and Schwartz, 2020; McAlpine et al., 2021). The publications by Afridi and Suk “*Neuroinflammatory basis of depression: learning from experimental models*,” and Hanslik et al. “*Modulation of glial function in health, aging, and neurodegenerative disease*,” provide up-to-date overviews on the physiological roles of microglia and astrocytes, and their involvement in the pathophysiology of certain diseases, like depression, Alzheimer's, and Parkinson's. These two review articles also offer very interesting insights from the authors, who underline some open questions and controversies in the field of glial cell biology and neurodegeneration.

Altogether, the manuscripts that constitute this Research Topic emphasize the need to advance our understanding about the mechanisms regulating the brain-immune axis, in order to develop effective therapeutic strategies and halt brain function decay in different neurological disorders.

## Author Contributions

All authors listed have made a substantial, direct, and intellectual contribution to the work and approved it for publication.

## Funding

KF is supported by the National Institutes of Health (R00 AG53412). AM is supported by the UK Dementia Research Institute (MRC, Alzheimer's Society, ARUK), the UKRI Medical Research Council (Career Development Award MR/V032488/1), and the French Foundation for Research on Alzheimer's Disease (FRA). AF is supported by the Portuguese Foundation for Science and Technology (2020.02753.CEECIND) and by the Swedish Research Council (grant no. 2019-02030). SD is supported by the Bright Focus Foundation (A2021025S), and the Cure Alzheimer's Fund.

## Conflict of Interest

The authors declare that the research was conducted in the absence of any commercial or financial relationships that could be construed as a potential conflict of interest.

## Publisher's Note

All claims expressed in this article are solely those of the authors and do not necessarily represent those of their affiliated organizations, or those of the publisher, the editors and the reviewers. Any product that may be evaluated in this article, or claim that may be made by its manufacturer, is not guaranteed or endorsed by the publisher.
